# Comparing protein–protein interaction networks of SARS-CoV-2 and (H1N1) influenza using topological features

**DOI:** 10.1038/s41598-022-08574-6

**Published:** 2022-04-07

**Authors:** Hakimeh Khojasteh, Alireza Khanteymoori, Mohammad Hossein Olyaee

**Affiliations:** 1 Department of Computer Engineering, University of Zanjan, Zanjan, Iran; 2 Department of Computer Engineering, Engineering Faculty, University of Gonabad, Zanjan, Gonabad, Iran

**Keywords:** Computational biology and bioinformatics, Machine learning, Network topology, Protein analysis

## Abstract

SARS-CoV-2 pandemic first emerged in late 2019 in China. It has since infected more than 298 million individuals and caused over 5 million deaths globally. The identification of essential proteins in a protein–protein interaction network (PPIN) is not only crucial in understanding the process of cellular life but also useful in drug discovery. There are many centrality measures to detect influential nodes in complex networks. Since SARS-CoV-2 and (H1N1) influenza PPINs pose 553 common human proteins. Analyzing influential proteins and comparing these networks together can be an effective step in helping biologists for drug-target prediction. We used 21 centrality measures on SARS-CoV-2 and (H1N1) influenza PPINs to identify essential proteins. We applied principal component analysis and unsupervised machine learning methods to reveal the most informative measures. Appealingly, some measures had a high level of contribution in comparison to others in both PPINs, namely Decay, Residual closeness, Markov, Degree, closeness (Latora), Barycenter, Closeness (Freeman), and Lin centralities. We also investigated some graph theory-based properties like the power law, exponential distribution, and robustness. Both PPINs tended to properties of scale-free networks that expose their nature of heterogeneity. Dimensionality reduction and unsupervised learning methods were so effective to uncover appropriate centrality measures.

## Introduction

SARS-CoV-2, a novel coronavirus mostly known as Covid-19, has become a matter of critical concern for every country around the world. It was first identified in December 2019 in Wuhan, China. The coronavirus Covid-19 has been affecting 220 countries and territories around the world. As of 7 January 2022, over 298 million cases have been confirmed cases and more than 5 million confirmed deaths attributed to the COVID-19 virus^1^.

Considering the high complexity of biological systems, one of the most challenging problems in experimental biology is designing a reliable experimental paradigm^2^. On the other hand, the aim of systems biology is to provide appropriate models with computational approaches using observational biological data, deposited in bioinformatics databases. These models are used for predicting purposes which in turn are useful for further experimental design^3^.

In the past several years, extensive experiments and data evolution have provided a good opportunity for systematic analysis and a comprehensive understanding of the topology of biological networks and biochemical processes in the cell^4^. In other words, we need to choose the right essential proteins to be targeted by new drugs^5^. However, identifying appropriate target proteins through experimental methods is time-consuming and expensive^5–7^. Both SARS-CoV-2 and (H1N1) influenza viruses have similar clinical symptoms^8^. Essential proteins play a vital role in the survival and development of the cell. They are also the most important materials in a variety of life processes. In cellular life, proteins are the chief actors that carry out the duties specified by the information encoded in genes^9^. The identification of essential proteins is decisive to understanding the minimal requirements for cellular life and practical purposes, such as a better understanding of diseases, and drug discovery^10^. Studying SARS-CoV-2 and (H1N1) influenza PPINs can be helpful to investigate similarities and differences between them. Studies have shown that protein–human protein interactions are biologically involved in multiple heterogeneous processes, including protein trafficking, translation, transcription, and regulation of ubiquitination^5,11^. For a more accurate understanding of their importance in cell life, it has to identify various interactions and determine the consequences of the interactions^12^. Moreover, this can use to empirically investigate complex network properties such as degree distribution^13^, power-law^14^, and other topological features.

Hahn et al.^15^ examined essential proteins in PPINs of eukaryotes: yeast, worm, and fly through three centrality measures. The results showed that there is a clear relationship between central proteins and survival. To detect which centrality measure is more suitable for choosing essential proteins in PPINs, Ernesto^16^ investigated the relationships between several centrality measures and subgraph centrality with essential proteins in the yeast PPIN. His study indicates that protein essentiality appears to be related to how much a protein is involved in clusters of proteins. As a result, subgraph centrality outperformed better than other measures for detecting essential proteins. Ashtiani et al.^17^ surveyed 27 centrality measures on yeast protein–protein interaction networks for ranking the nodes in all PPINs. They examined the correlation between centrality measures through unsupervised machine learning methods.

Although, in the context of analyzing PPINs, the comparison of different networks is challenging. There are various gene profiling for SARS-CoV-2 and (H1N1) influenza in the GenBank database^18,19^. Unfortunately, it has not been done APMS (affinity purification coupled to mass spectrometry) for building corresponding PPINs for most of them. These experimental procedures require considerable time and resources. In this work, we adopt the human protein–protein interaction (PPI) data set from^20,21^ database to compare SARS-CoV-2 and (H1N1) influenza PPINs. Using these networks, we then analyze the topological features, focusing on the properties of the graphs which represent these networks. We consider some specific measures, such as graph density, degree distribution, and 21 different centrality measures. We fit power law and exponential distributions on these networks and calculate alpha power and R-squared values.

## Materials and methods

### Materials

There are four different types of Coronaviruses (CoVs) includes Alphacoronoavirus, Betacoronavirus, Deltacoronavirus, and Gammacoronavirus^20^. Betacoronavirus includes five subtypes among Embecovirus, Sarbecovirus, Merbecovirus, Nobecovirus, and Hibecovirus. SARS-CoV and SARS-CoV-2 are from Sarbecovirus (SV) subgenus. Khorsand et al.^20^ created a Sarbecovirus-human protein–protein interaction network. We have derived SARS-CoV-2 PPINs from this dataset. For (H1N1) influenza PPIN, Khorsand et al.^21^ made Comprehensive PPINs for all genres of Alphainfluenza viruses (IAV). The main human influenza pathogens are Alphainfluenza viruses (IAV) that include subtypes of combining one of the 16 hemagglutinin (HA: H1–H16) with one of the 9 neuraminidase (NA: N1–N9) surface antigens. We have downloaded the whole network and separated (H1N1) influenza PPIN from the Alphainfluenza protein–protein interaction network. SARS-CoV-2 PPIN contains 1922 interactions between 14 SARS-CoV-2 proteins and 1395 human proteins and (H1N1) influenza PPIN contains 9174 interactions between 46 (H1N1) influenza proteins and 2751 human proteins.

### Methods

We propose a useful analysis approach to compare SARS-CoV-2 and (H1N1) influenza PPINs. At first, we need to select a valid dataset and so, investigate and select suitable features that are meaningful in a biological system. Next, we develop our approach to make comparisons and the results are analyzed. In the following, we describe how to deal with these phases, respectively. The process starts by computing global network properties. In the next phase, 21 different centrality measures are applied to both networks, standard normalization and PCA are used on centrality values, respectively. Using some machine learning methods, the centrality measures are compared and analyzed.

### Network Global properties

In this study, we have considered some of the network properties such as graph density, graph diameter, and centralization. In the following, we review these network concepts. All these properties are calculated and analyzed in both networks using igraph^22^ R package. Then, the power-law distribution is checked out by computing α and R-squared values. R-squared is the percentage of the response variable variation that is described by a linear model^23^.

Although, PPINs are directed but most of analyzing methods consider PPINs as undirected^24,25^. For this research study, we considered PPINs as undirected and loop-free connected graphs. So, let $G = \left( {V, E} \right)$ be an undirected graph. This graph consists of nodes represented by $V = \left\{ {v_{1},v_{2} , \ldots } \right\}$ and edges $E = \left\{ {e_{1} ,e_{2} , \ldots } \right\}$ such that any edge $e_{ij} \in E$ represents the connection between nodes $v_{i}$ and $v_{j} \in V$.

### Graph density

The density of a graph is the fraction of the number of edges to the number of possible edges^26^. Density is equal to $2*\left| E \right|$ divided by |$V|*\left( {\left| V \right| - 1} \right)$. A complete graph has density 1; the minimal density of any graph is 0. There are some features for identifying biological networks. Often, biological networks are incomplete or heterogeneous which means very low density^27^.

### Graph diameter

In a network, diameter is the longest shortest path between any two vertices $\left( {u,v} \right)$, where *d* $\left( {u,v} \right)$ is a graph distance^28^.

### Heterogeneity

The network heterogeneity is defined as the coefficient of variation of the connectivity distribution:

1 $$ {\text{Heterogeneity}} = \frac{{\sqrt {variance\left( k \right)} }}{mean\left( k \right)} $$

In PPINs, the connectivity $k_{i}$ of node $i$ equals the number of directly linked neighbors. PPINs tend to be very heterogeneous. Highly connected 'hub' nodes in PPINs have an important role in the network. A hub protein is essential and contains many distinct binding sites to accommodate non-hub proteins^29^.

### Centralization

Centralization is a method that gives information about the topology of a network. Centralization is measured from the centrality scores of the vertices. The centralization that closes to 1, illustrates that probably the network has a star-like topology. If it is closer to 0, the more likely topology of the network is like square whereas every node of the network has at least 2 neighbors)^28^. This metric is calculated as follows^30^:

2 $$ C_{x} = \frac{{\mathop \sum \nolimits_{i = 1}^{N} [C_{x} \left( {p_{*} } \right) - C_{x} \left( {p_{i} } \right)]}}{{\max \mathop \sum \nolimits_{i = 1}^{N} [C_{x} \left( {p_{*} } \right) - C_{x} \left( {p_{i} } \right)]}} $$

where $$C_{x} \left( {p_{i} } \right)$$ is any centrality measure of point $$ i$$ and $$C_{x} \left( {p_{i*} } \right)$$ is the largest such measure in the network. Each centrality measure can be used (betweenness centrality, closeness centrality and etc.).

### Centrality analysis

In this work, the following 21 centrality measures are selected: Average Distance^31^, Barycenter^32^, Closeness (Freeman)^30^, Closeness (Latora)^33^, Residual closeness^34^, Decay^35^, Diffusion degree^36^, Geodesic K-Path^37,38^, Laplacian^39^, Leverage^40^, Lin^41^, Lobby^42^, Markov^43^, Radiality^44^, Eigenvector^45^, Subgraph scores^16^, Shortest-Paths betweenness^30^, Eccentricity^46^, Degree^28^, Kleinberg’s authority scores^47^, and Kleinberg’s hub scores^47^. These measures are calculated using the centiserve^48^ and igraph^22^ R packages. We have classified the centrality measures into five distinct classes including Distance-, Degree-, Eigen-, Neighborhood-based and Miscellaneous groups depend on their logic and formulas (Table 1). Tables 2 and 3 show the definitions for 21 different centrality measures based on their group.

**Table 1:** Centrality measures. The centrality measures are classified in five groups depending on their logic and formula.

Distance based	Degree based	Eigen based	Neighborhood based	Miscellaneous
Average Distance	Kleinberg’s authority centrality scores	Eigenvector Centrality Scores	Subgraph centrality scores	Geodesic K-Path Centrality
Barycenter	Degree Centrality	Laplacian Centrality		Markov Centrality
Closeness Centrality (Freeman)	Diffusion Degree			Shortest-Paths Betweenness Centrality
Closeness Centrality (Latora)	Kleinberg’s hub centrality scores			
Decay Centrality	Leverage Centrality			
Eccentricity	Lobby Index (Centrality)			
Lin Centrality				
Radiality Centrality				
Residual Closeness centrality

**Table 2:** Definitions for distance based centrality measures.

Centrality	Formula	Description	References
**Distance based**			
Average Distance	$C_{u} = \frac{{\mathop \sum \nolimits_{w \in V} dist\left( {u,w} \right)}}{n - 1}$	Average distance of node $u$ to the rest of nodes in the net	^28,31^
Barycenter	$C_{u} = \frac{1}{{\mathop \sum \nolimits_{w \in V} dist\left( {u,w} \right)}}$	Inverse of total distance from $u$ to all other vertices	^32^
Closeness Centrality (Freeman)	$C_{u} = \frac{1}{{\mathop \sum \nolimits_{{w \in V\backslash \left\{ u \right\}}} dist\left( {u,w} \right)}}$	Inverse of average distance	^30^
Closeness Centrality (Latora) Or Harmonic centrality	$C_{u} = \mathop \sum \limits_{u \ne w \in V} \frac{1}{{dist\left( {u,w} \right)}}$	The sum of inverse of the distance from $u$ to all other vertices	^33^
Decay Centrality	$\mathop \sum \limits_{w \in V} \delta^{{dist\left( {u,w} \right)}}$	Where $dist\left( {u,w} \right)$ denotes the distance between $u$ and $w$ and $\delta \in \left( {0, 1} \right)$ is a parameter	^35^
Eccentricity	$C_{u} = {\text{max}}\left\{ {dist\left( {u,w} \right):w \in V} \right\}$	The distance between node $u$ and the most distant node in the net	^46^
Lin Centrality	$C_{u} = \frac{{\left| {\left\{ {w{|}dist\left( {w,u} \right) < \infty } \right\}} \right|^{2} }}{{\mathop \sum \nolimits_{{dist\left( {w,u} \right) < \infty }} dist\left( {w,u} \right)}}$		^41^
Radiality Centrality	$C_{u} = \frac{{\mathop \sum \nolimits_{w \in V} \left( {diamG + 1 - dist\left( {u,w} \right)} \right)}}{n - 1}$	The easiness of reaching any node from node $u$	^44^
Residual Closeness centrality	$$C_{u} = \mathop \sum \limits_{w} \mathop \sum \limits_{t \ne w} \frac{1}{{2^{{d_{u} \left( {w,t} \right)}} }}$$	Let $$d_{u} \left( {w,t} \right)$$ be the distance between vertices $$w$$ and $$ t$$ in the graph, received from the original graph where all links of vertex $$u$$ are deleted	^34^

**Table 3:** Definitions for Degree based, Eigen based, Neighborhood based, and Miscellaneous centrality measures.

Centrality	Formula	Description	References
**Degree based**			
Degree Centrality	$C_{u} = k\left( u \right)$	Degree of node $u$	^28^
Diffusion Degree	$C_{D} \left( v \right) = \mathop \sum \limits_{i = 1}^{n} \sigma (u_{i } ,v)$	Where function $\sigma \left( {u_{i} ,v} \right)$ defined as, $\sigma \left( {u_{i} ,v} \right) = 1$ if and only if $u_{{i}}$ and $v$ are connected and $\sigma \left( {u_{i} ,v} \right) = 0$ otherwise	^36^
Kleinberg’s authority centrality scores	$auth\left( p \right) = \mathop \sum \limits_{{q \in P_{to} }} hub\left( q \right)$	Where $P_{to}$ is all pages which link to page $p$. That is, a page's authority score is the sum of all the hub scores of pages that point to it	^47^
Kleinberg’s hub centrality scores	$hub\left( p \right) = \mathop \sum \limits_{{q \in P_{from} }} auth\left( q \right)$	Where $P_{from}$ is all pages which page $p$ links to. That is, a page's hub score is the sum of all the authority scores of pages it points to	^47^
Leverage Centrality	$l_{i} = \frac{1}{{k_{i} }}\mathop \sum \limits_{{N_{i} }} \frac{{k_{i} - k_{j} }}{{k_{i} + k_{j} }}$	Leverage centrality is a measure of the relationship between the degree of a given node ($k_{i}$) and the degree of each of its neighbors ($k_{j}$), averaged over all neighbors ($N_{i}$)	^40^
Lobby Index (Centrality)		The lobby index of a node x is the largest integer k such that x has at least k neighbors with a degree of at least k	^42^
**Eigen based**			
Eigenvector Centrality Scores	$C_{u} = \frac{1}{\lambda }\mathop \sum \limits_{t \in V} a_{v,t} C_{t}$	Let $a_{v,t}$ be the adjacency matrix	^45^
Laplacian Centrality	$$C_{v}^{L} = d_{G}^{2} \left( v \right) + d_{G} \left( v \right) + 2\mathop \sum \limits_{{v_{i} \in N\left( v \right)}} d_{G} \left( {v_{i} } \right)$$	Where $$G$$ is a graph of $$n$$ vertices, $$N\left( v \right)$$ is the set of neighbors of $$ v$$ in $$G$$ and $$d_{G} \left( {v_{i} } \right) $$ is the degree of $$v_{i}$$ in $$G$$	^39^
**Neighborhood based**			
Subgraph centrality scores	$SC\left( v \right) = \mathop \sum \limits_{k = 0}^{\infty } \frac{{\mu_{k} \left( v \right)}}{k!}$	The number of closed walks of length $k$ starting and ending node $v$ in the network is given by the local spectral moments $\mu_{k} \left( v \right).$	^49^
**Miscellaneous**			
Geodesic K-Path Centrality	$C^{k} \left( v \right) = \mathop \sum \limits_{s \in V} \frac{{\sigma_{s}^{k} \left( v \right)}}{{\sigma_{s}^{k} }}$	Where $s$ are all the possible source nodes, $\sigma_{s}^{k} \left( v \right)$ is the number of κ-paths originating from s and passing through $v$ and $\sigma_{s}^{k}$ is the overall number of κ-paths originating from $s$	^37,38^
Markov Centrality	$$C_{M} \left( v \right) = \frac{n}{{\mathop \sum \nolimits_{s \in V} m_{sv} }}$$	The Markov centrality index $$C_{M} \left( v \right)$$ uses the inverse of the average MFPTs to define the importance of node $$v$$ where $$n = \left| R \right|$$, $$R$$ is a given root set, and $$m_{st} $$ is the MFPT from $$s$$ to $$t$$	^43^
Betweenness Centrality	$C_{B} \left( v \right) = \mathop \sum \limits_{s \ne v \ne t} \frac{{\sigma_{st} \left( v \right)}}{{\sigma_{st} }}$	Where $\sigma_{st}$ is the total number of shortest paths from node $s$ to node $t$ and $\sigma_{st} \left( v \right)$ is the number of those paths that pass through $v$	^30^

### Unsupervised machine learning analysis

principal component analysis (PCA) is a dimensionality-reduction method that is often used to reduce the dimensionality of large data sets, by linear transforming a large set of variables into smaller ones^50^. PCA aims to remove correlated centralities, reduce overfitting, and better visualization. Since the values of centrality measures are in different scales and PCA is affected by scale, Standard normalization has been undertaken on centrality measures before applying PCA. This phase is significant because it helps to recognize which centrality measures can determine influence nodes within a network. Then, PCA is used on normalized computed centrality measures. In the next phase, it is assessed that whether it is feasible to cluster the centrality measures in both networks according to clustering tendency. Before applying any clustering method to the dataset, it is important to evaluate whether the data sets contain meaningful clusters or not. For assessment of the feasibility of the clustering analysis, the Hopkins’ statistic values and visualizing VAT (Visual Assessment of Cluster Tendency) plots are calculated by factoextra R package^51^. Some validation measures are used to select the most suitable clustering method among hierarchical, k-means, and PAM (Partitioning Around Medoids) methods using the clValid package^52^. In this study, we apply Silhouette scores to select the appropriate method. After the choice of the clustering method, factoextra package is employed to find the optimal number of clusters^51^. In the clustering procedure, Ward’s Method^53^ is used as a dissimilarity measure. Ward’s minimum variance method creates groups such that variance is minimized within clusters.

## Results and discussions

### Evaluation of network properties

**Figure 1:**
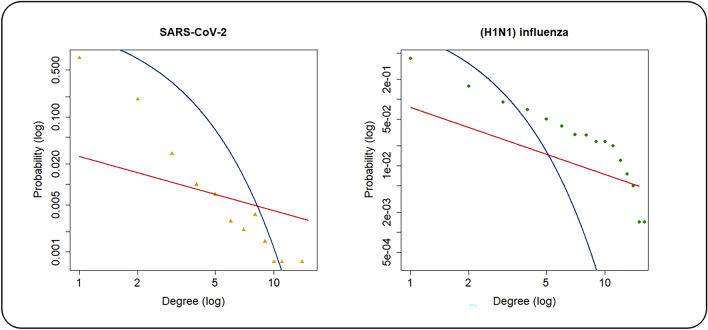
Fitting both SARS-CoV-2 and (H1N1) influenza PPINs on power-law distribution.

In this study, both networks were examined to compare global properties. The network global properties were computed for both networks (Table 4). Firstly, we compared the networks based on their nodes. We realized that SARS-CoV-2 and (H1N1) influenza PPINs include 553 common human proteins. The list of these proteins is available and provided as supplementary material (Supplementary File 1). The densities of SARS-CoV-2 and (H1N1) influenza PPINs were computed at 0.0019 and 0.0023 that was expected because biological networks are usually sparse. The network diameters were equal in both networks. SARS-CoV-2 and (H1N1) influenza PPINs were correlated to the power-law distribution with high alpha power and R-squared values. In terms of comparison of heterogeneity values, SARS-CoV-2 PPIN achieved a higher value. But, both networks are relatively heterogeneous. The heterogeneous network exhibits many unique properties of scale-free networks^54^. Values of network centralization were very close together. Figure 1 demonstrates power law (red curve) and exponential (blue curve) distributions in SARS-CoV-2 and (H1N1) influenza PPINs. Both the degree distributions were left-skewed analogous to scale-free networks.

**Table 4:** Network global properties of SARS-CoV-2 and (H1N1) influenza PPINs.

Networks Properties	Nodes	Edges	Density	Diameter	α value (Power Law)	R-squared (Power Law)	Heterogeneity	Network Centralization
SARS-CoV-2	1409	1922	0.0019	6	0.805	0.54	7.3628	0.3089
(H1N1) influenza	2797	9174	0.0023	6	1.009	0.717	5.3197	0.2392

### Centrality analysis

**Figure 2:**
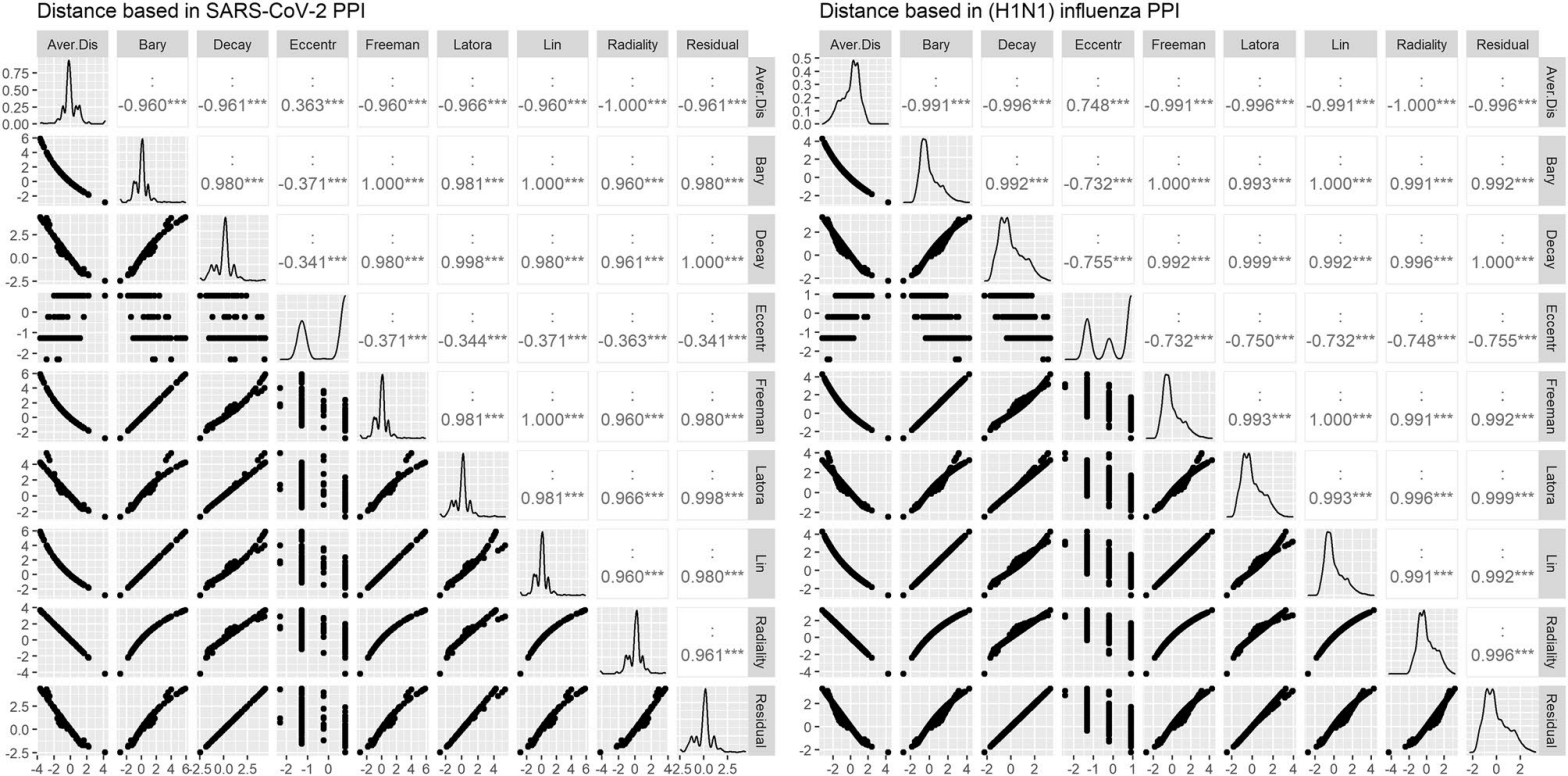
r Pearson correlation coefficients between centralities in the group of Distance based and pairwise scatter plots of centrality measures.

**Figure 3:**
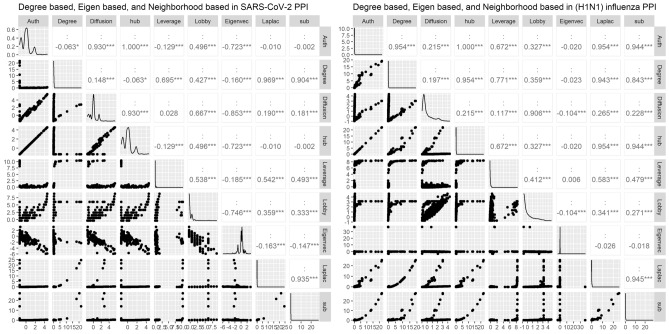
r Pearson correlation coefficients between centralities in the group of Degree based, Eigen based, and Neighborhood based and pairwise scatter plots of centrality measures.

**Figure 4:**
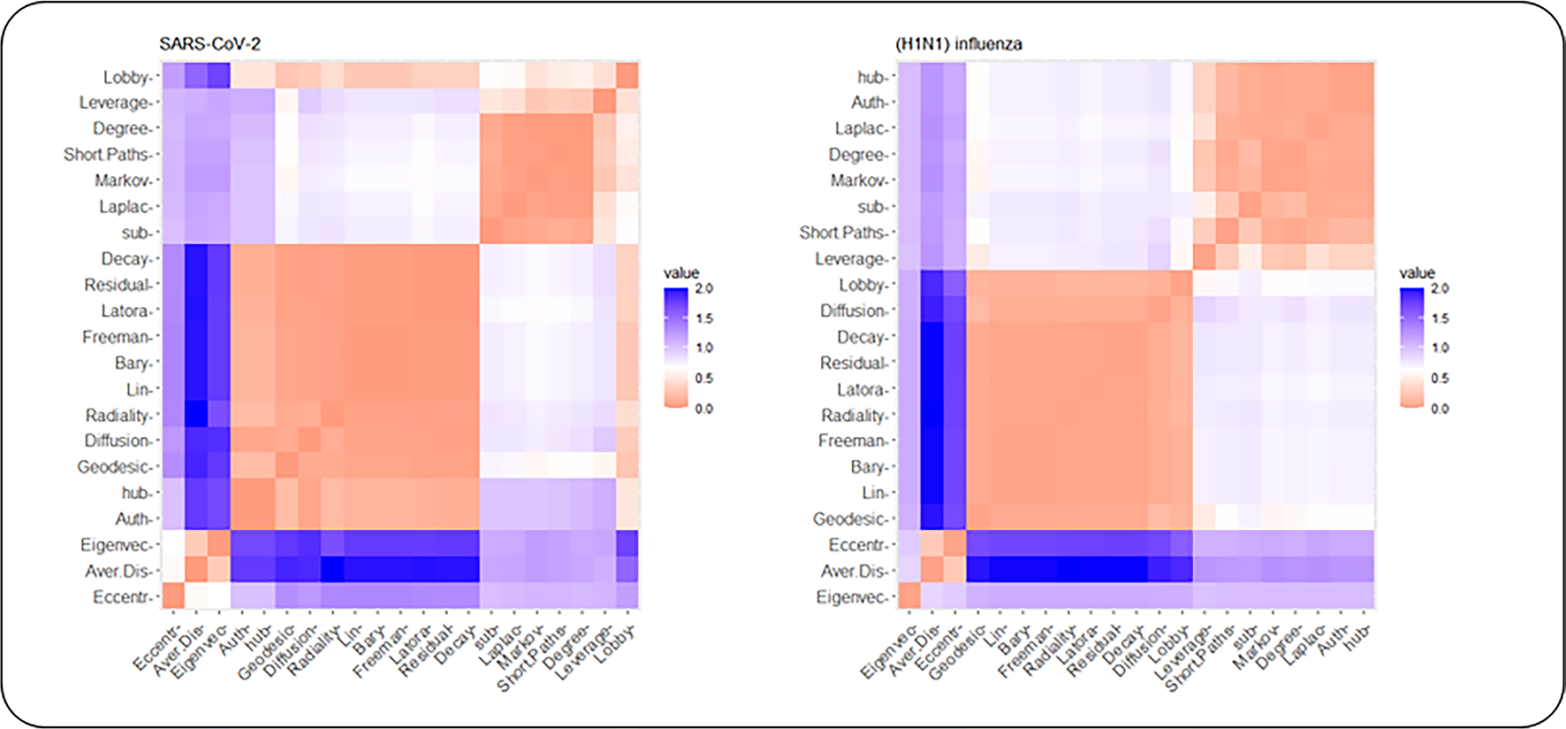
The dissimilarity matrix based on the Pearson correlation coefficient for all centrality measures in both networks.

In the next phase, the 21 centrality measures of nodes were calculated in both networks. The centrality measures were divided into two groups according to Table 2: (1) Distance based and (2) Degree based, Eigen based, and Neighborhood based. The top 10 essential proteins identified by 21 centrality measures in PPINs are given in as supplementary material (Supplementary File 2) for experimental validation. The r Pearson correlation coefficients between centralities in two groups and pairwise scatter plots of centrality measures were also shown in Figs. 2 and 3. These plots illustrate that there is a clear correlation in some of the centrality measures. For a better comparison, we also provided the dissimilarity matrix based on the Pearson correlation coefficient for all centrality measures in both networks (Fig. 4). The Pearson correlation coefficient puts within the range [− 1,1]. In some applications, such as clustering, it can be reasonable to transform the correlation coefficient to a dissimilarity measure^52^. In this way, the Pearson distance lies in the interval [0,2]. A value of 0 indicates that would not be a correlation between the two centrality measures. The higher value demonstrates the more correlation between them. In both networks, the matrixes indicate a high positive association between Average Distance and Radiality centrality measures are highly associated together. Furthermore, in (H1N1) influenza, these correlations are more clear between Average Distance and Lin, Barycenter, Closeness (Freeman), Radiality, Closeness (Latora), Residual closeness, and Decay measures.

### Dimensionality reduction and clustering analysis

**Figure 5:**
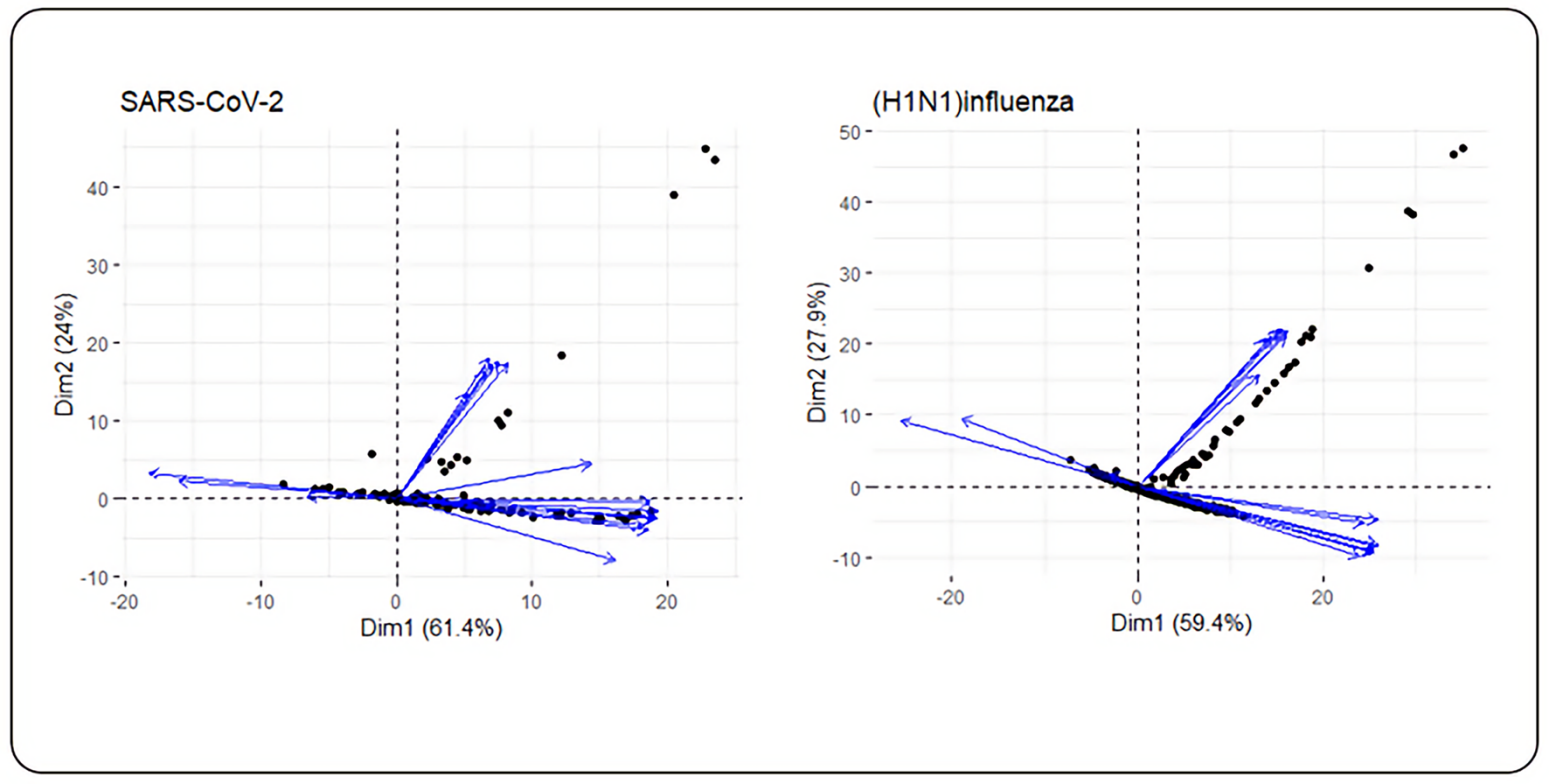
Biplot representation of the centrality measures in SARS-CoV-2 and (H1N1) influenza PPINs. In each plot, nodes were shown as points and centrality measures as vectors.

**Figure 6:**
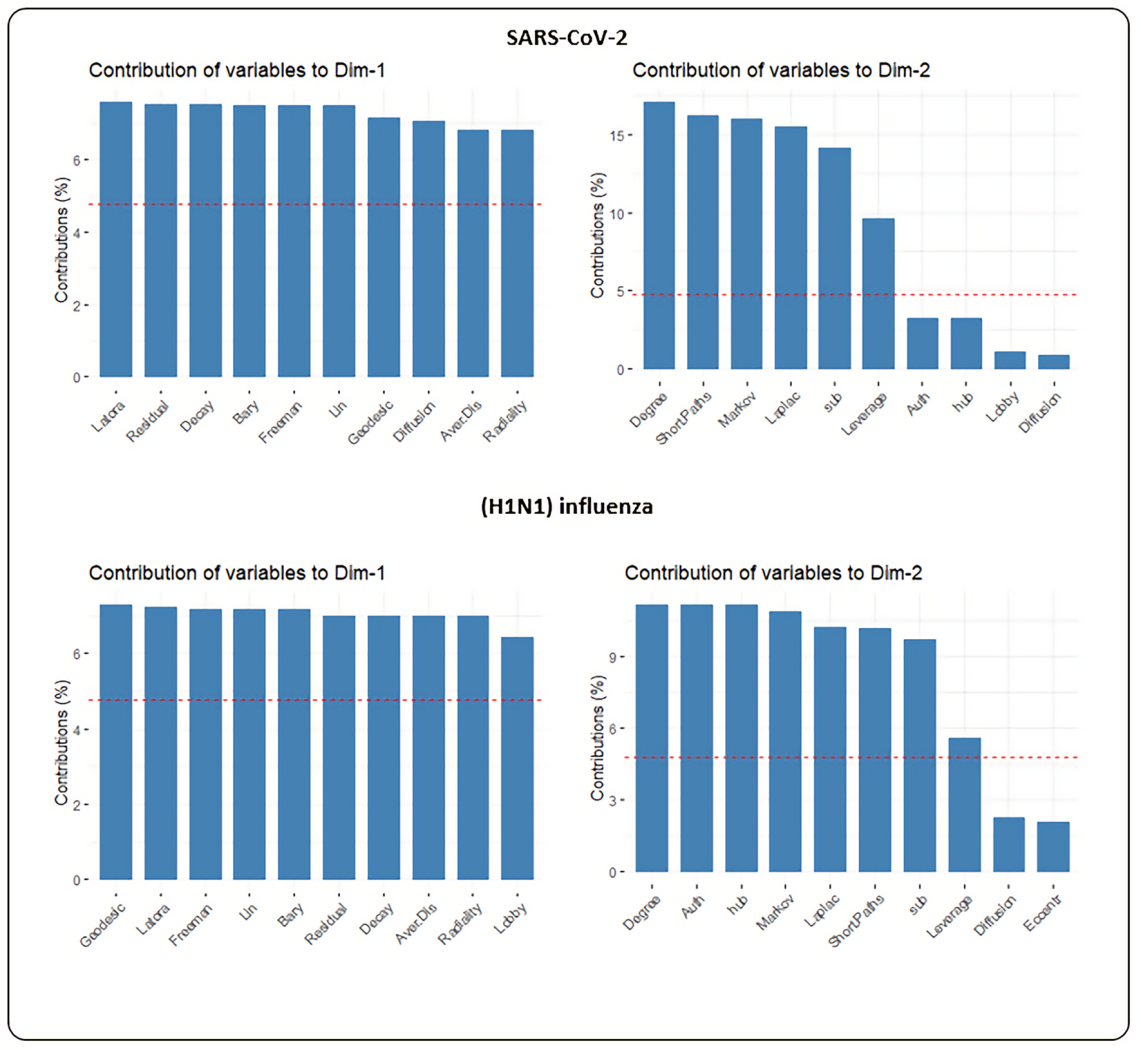
The top 10 centrality measures contributing to PCA for two dimensions.

In the next phase, PCA-based dimensionality reduction was applied to centrality measures to show a visual representation of the dominant centrality measures in the data set. The profile of the distance to the center of the plots and their directions were mostly harmonic for both networks as illustrated in Fig. 5. The contribution of each centrality measure for two dimensions is given as supplementary material (Supplementary File 3). The percentage of contribution of variables (i.e. centrality measures) in a given PC was computed as (variable. Cos2*100)/(total Cos2 of the component)). Figure 6 illustrates the first ten contributing centrality measures to PCA for two dimensions. In both networks, the contribution percent for the first ten contributors is too close for the first dimension. For the second dimension, degree centrality is the major contributor for both PPINs. Eigenvector and Eccentricity revealed a low contribution value in both PPINs. In contrast, Closeness (Latora) displayed high levels of contribution in both networks whilst it was the first rank of SARS-CoV-2 PPIN contributors and second rank of (H1N1) influenza PPIN contributors. Also, we have acquired the contribution of each centrality measure for two dimensions sorted by the p-value of the correlation (Supplementary File 4 and 5). The significance level in this study was considered equal to 0.05. A lower p-value in the results exhibits a strong relationship between centrality measures in both networks.

**Figure 7:**
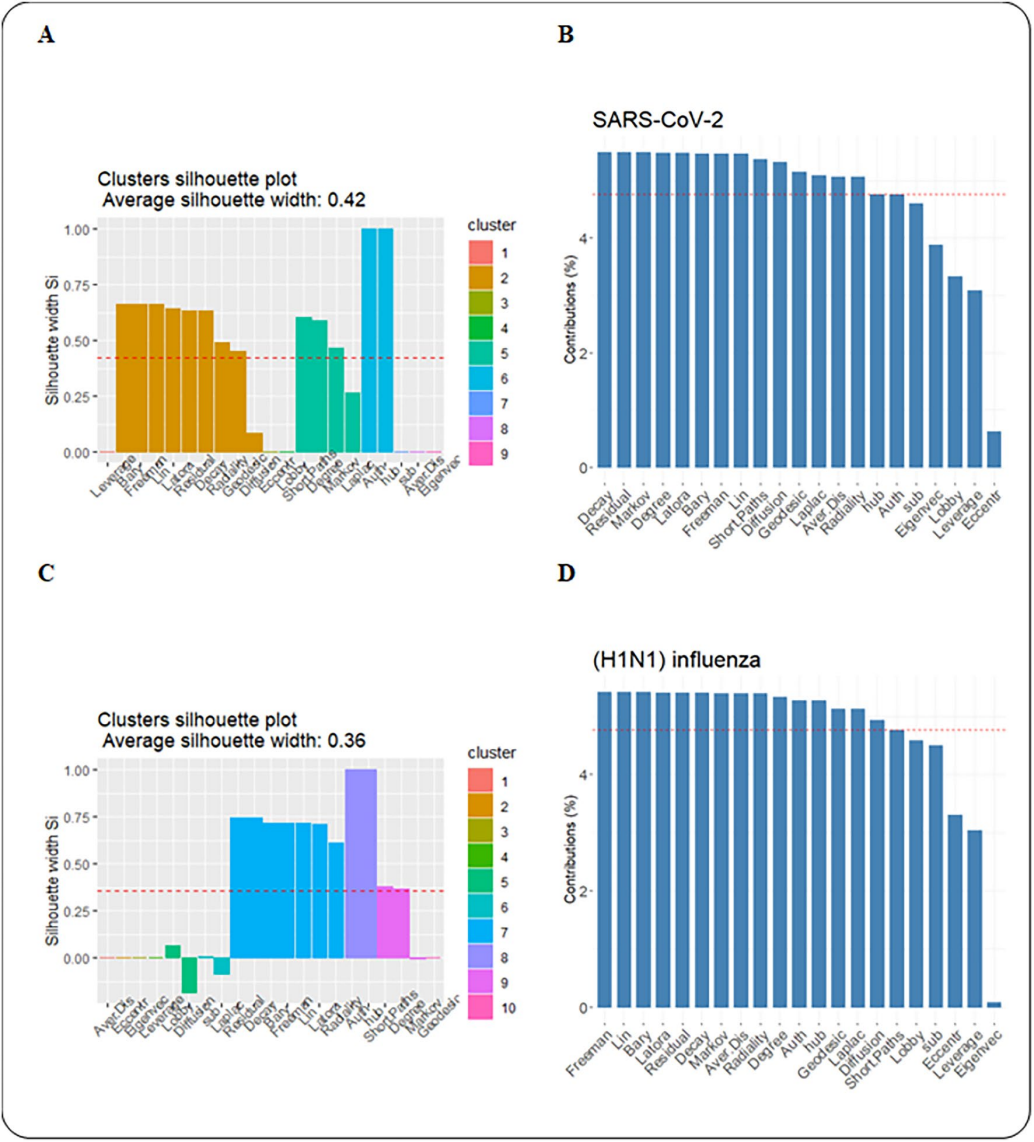
(**A**) Clustering silhouette plot of the combined-score PPIN. The colors represented the nine clusters of the centrality measures in SARS-CoV-2 PPIN. The average silhouette width was 0.42. (**B**) Contribution values of centrality measures according to their corresponding principal components in SARS-CoV-2 PPIN. (**C**) Clustering silhouette plot of the combined-score PPIN. The colors represented the ten clusters of the centrality measures in (H1N1) influenza. The average silhouette width was 0.36. (**D**) Contribution values of centrality measures according to their corresponding principal components in (H1N1) influenza PPIN.

**Figure 8:**
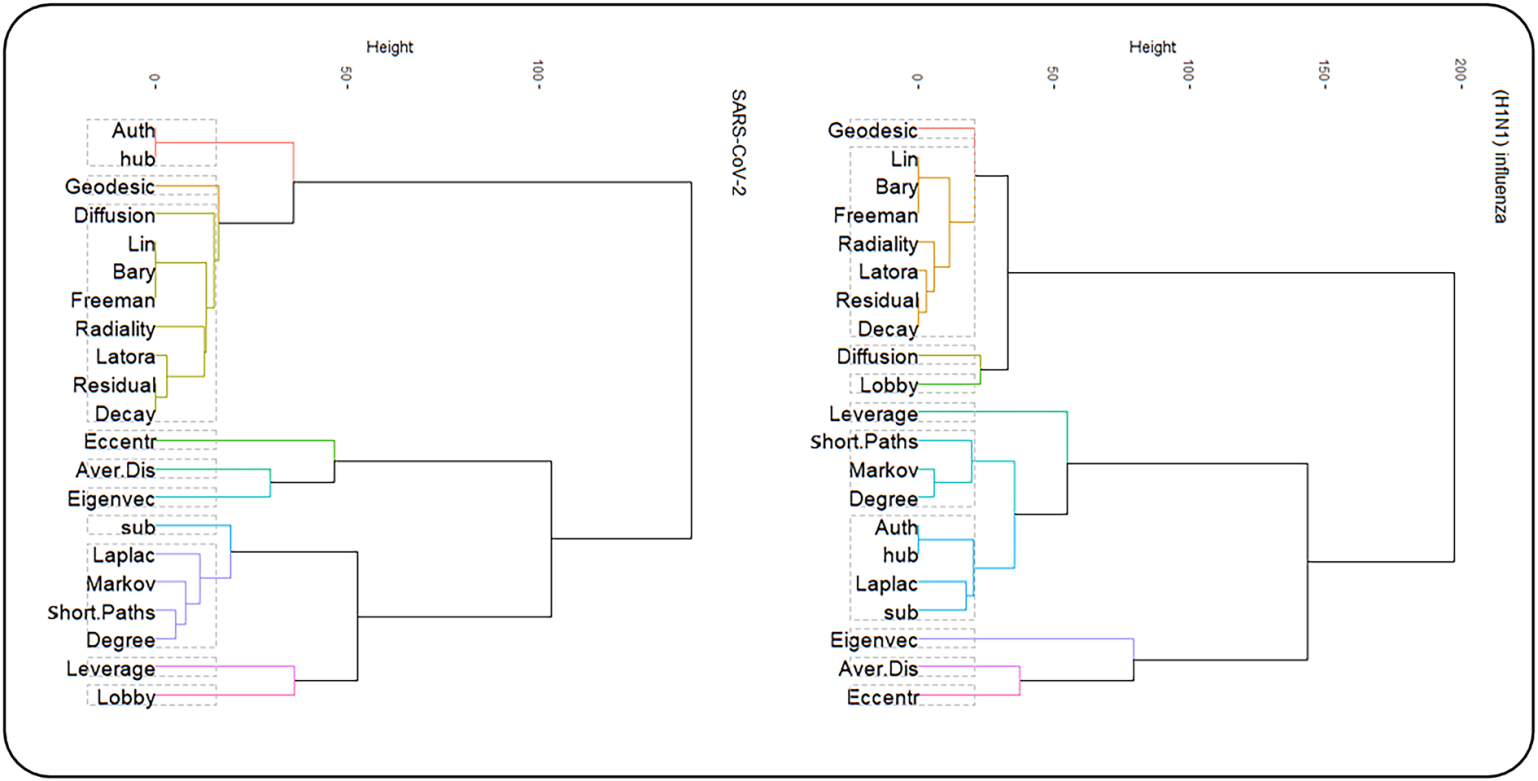
Clustering dendrograms for SARS-CoV-2 and (H1N1) influenza PPINs.

Ultimately, we performed unsupervised classification to cluster centrality values computed in PPINs. First, we executed a clustering tendency procedure. For clustering centrality values in each network, we considered Hopkins statistics were more than the threshold. The threshold value was 0.05^17^. The results are provided in the first column of Table 5 and supplementary material (Supplementary File 6). Then, silhouette scores were calculated in three methods (i.e. hierarchical, k-means, and PAM) and average Silhouette width were evaluated in clustering the data sets. These scores are available and provided as supplementary material (Supplementary File 7). Finally, based on average Silhouette width, the k-means method was selected for clustering centrality values in both PPINs (Fig. 7). The outputs of the clustering method and the corresponding number of clusters were also shown in Table 5. The optimal number of clusters was also determined by k-means and PAM clustering algorithms. These results are given as supplementary material (Supplementary File 8). The centrality measures were clustered in each PPINs using the hierarchical algorithm based on Ward’s method^50^ that was shown in Fig. 8.

**Table 5:** Clustering information values for PPINs.

Network	Hopkins Statistic	Number of Clusters	Average Silhouette width
SARS-CoV-2	0.75	9	0.42
(H1N1) influenza	0.77	10	0.36

## Discussion

At the validation step, we encountered remarkable results. Silhouette scores of centrality measures illustrated the centrality measures in the same clusters had very close contribution values for these measures (Fig. 7). In SARS-CoV-2 PPIN, Barycenter, Decay, Diffusion degree, Closeness (Freeman), Geodesic K-Path, Closeness (Latora), Lin, Radiality, and Residual closeness measures were in the same cluster. Also, in (H1N1) influenza, Barycenter, Decay, Closeness (Freeman), Closeness (Latora), Lin, Radiality, and Residual closeness were measures were in the same cluster. The average silhouette scores were 0.55 and 0.71 in these clusters for SARS-CoV-2 and (H1N1) influenza PPINs, respectively. The centrality measures namely Shortest-Paths betweenness, Laplacian, Degree, and Markov measures were in a cluster for SARS-CoV-2 PPIN where the mean of their silhouette scores (i.e. 0.48) was higher than the overall average, and in the same way, their corresponding contribution values were high, too. Kleinberg’s hub and Kleinberg’s authority scores are grouped in a cluster in both PPINs and their corresponding contribution values were equal.

Our results demonstrated that an exclusive profile of centrality measures including Barycenter, Decay, Closeness (Freeman), Closeness (Latora), Lin, Radiality, and Residual closeness was the most significant index to determine essential nodes. We inferred that both PPINs have close results in centrality analysis. Also, our research confirmed an analogous study^17^ about the relationship between contribution value derived from PCA and silhouette width as a cluster validation. Furthermore, our centrality analysis resulted in many equal values in all centrality measures that imply dynamic robustness in PPINs. Also, it reveals that PPINs due to sparsity and tree-like topology are more explorable than random networks with higher connectivity^55^.

## Conclusion

SARS-CoV-2, a novel coronavirus mostly known as COVID-19, has become a matter of critical concern around the world. Besides, network-based methods have emerged to analyze, and understand complex behavior in biological systems with a focus on topological features. In recent decades, network-based ranking methods have provided systematic analysis for predicting influence proteins and proposing drug target candidates in the treatment of types of cancer and biomarker discovery. SARS-CoV-2 and (H1N1) influenza PPINs have 553 common human proteins. Studying and comparing these networks can be an effective step to identify new drug compounds for biological targets.

In this study, we have analyzed SARS-CoV-2 and (H1N1) influenza PPINs topologically. We employed heterogeneity measure to PPINs. The heterogeneity results and fitting distributions demonstrated the properties of scale-free networks in both networks. Subsequently, 21 centrality measures were utilized to prioritize the proteins in both networks. We illustrated that dimensionality reduction methods like PCA can help to extract more relevant features (i.e. centrality measures) and corresponding relationships in unsupervised machine learning methods. Thus, to detect influential nodes in biological networks, PCA can help to select suitable measures. In other words, dimensionality reduction methods can illuminate which measures have the highest contribution values, i.e., which measures contain much more useful information about centrality.

## Supplementary Information

Supplementary Information 1.

Supplementary Information 2.

Supplementary Information 3.

Supplementary Information 4.

Supplementary Information 5.

Supplementary Information 6.

Supplementary Information 7.

Supplementary Information 8.

## Data Availability

All the data and materials used in this paper are available at: https://github.com/Khojasteh-hb/Comparing-PPI-networks-of-SARS-CoV-2-and-H1N1-influenza.
