# Supplementary material for: Comparing protein–protein interaction networks of SARS-CoV-2 and (H1N1) influenza using topological features
Source: Sci Rep. 2022 Apr 7;12:5867. doi: 10.1038/s41598-022-08574-6 (PMC8988119; doi:10.1038/s41598-022-08574-6)
Supplement: Supplementary file 3 — Supplementary Information 3. [file 41598_2022_8574_MOESM3_ESM.pdf]

The contribution of each centrality measure to PCA for each network

| Centralities \ Networks | SARS-CoV-2 | | (H1N1) influenza | |
|---|---|---|---|---|
| | Dim.1 | Dim.2 | Dim.1 | Dim.2 |
| **Average Distance** | 6.82 | 0.58 | 7 | 1.94 |
| **Barycenter** | 7.49 | 0.29 | 7.17 | 1.68 |
| **Closeness Centrality (Freeman)** | 7.49 | 0.29 | 7.17 | 1.68 |
| **Closeness Centrality (Latora)** | 7.58 | 0.13 | 7.22 | 1.55 |
| **Residual Closeness centrality** | 7.52 | 0.35 | 7.01 | 2 |
| **Decay Centrality** | 7.52 | 0.35 | 7.01 | 2 |
| **Diffusion Degree** | 7.06 | 0.88 | 6.2 | 2.23 |
| **Geodesic K-Path Centrality** | 7.17 | 0.01 | 7.28 | 0.54 |
| **Laplacian Centrality** | 1.02 | 15.49 | 2.73 | 10.22 |
| **Leverage Centrality** | 0.55 | 9.59 | 1.58 | 5.59 |
| **Lin Centrality** | 7.49 | 0.29 | 7.17 | 1.68 |
| **Lobby Index (Centrality)** | 4.22 | 1.07 | 6.43 | 0.64 |
| **Markov Centrality** | 1.38 | 16 | 2.82 | 10.88 |
| **Radiality Centrality** | 6.82 | 0.58 | 7 | 1.94 |
| **Eigenvector Centrality Scores** | 5.29 | 0.27 | 0.1 | 0.06 |
| **Subgraph centrality scores** | 0.89 | 14.1 | 2.07 | 9.7 |
| **Shortest-Paths Betweenness Centrality** | 1.15 | 16.17 | 2.23 | 10.17 |
| **Eccentricity** | 0.88 | 0.01 | 3.88 | 2.08 |
| **Degree Centrality** | 0.96 | 17.06 | 2.61 | 11.15 |
| **Kleinberg’s authority centrality scores** | 5.36 | 3.25 | 2.52 | 11.14 |
| **Kleinberg’s hub centrality scores** | 5.36 | 3.25 | 2.52 | 11.14 |
