# Supplementary figures and images for: Comparing protein–protein interaction networks of SARS-CoV-2 and (H1N1) influenza using topological features
Source: Sci Rep. 2022 Apr 7;12:5867. doi: 10.1038/s41598-022-08574-6 (PMC8988119; doi:10.1038/s41598-022-08574-6)

## Hopkins statistics

SARS-CoV-2

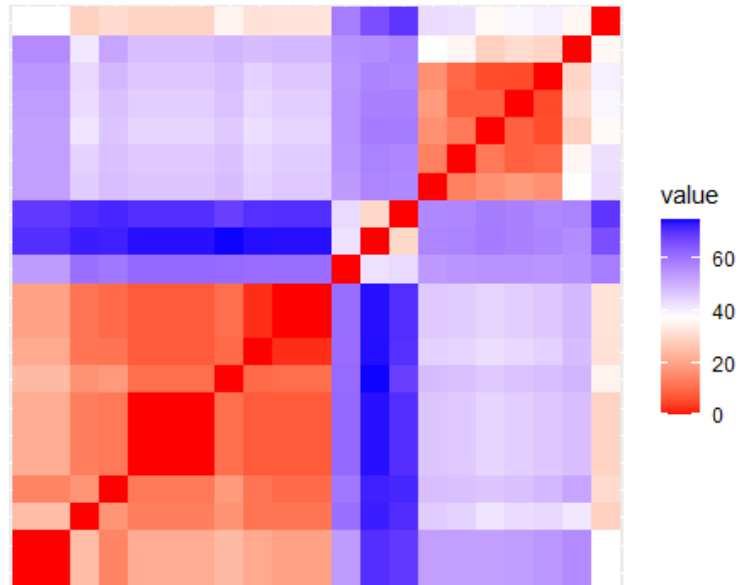

(H1N1) influenza

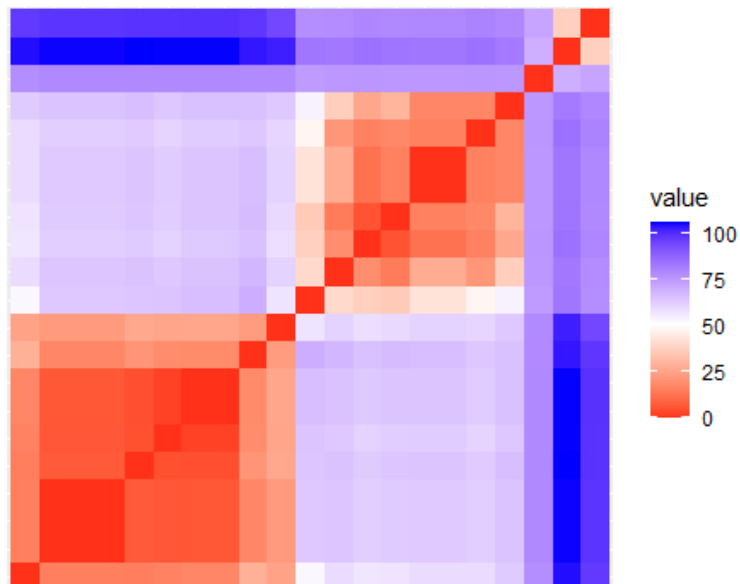

Supplement: Supplementary file 6 — Supplementary Information 6. [file 41598_2022_8574_MOESM6_ESM.pdf]

## Optimal number of clusters

### SARS-CoV-2

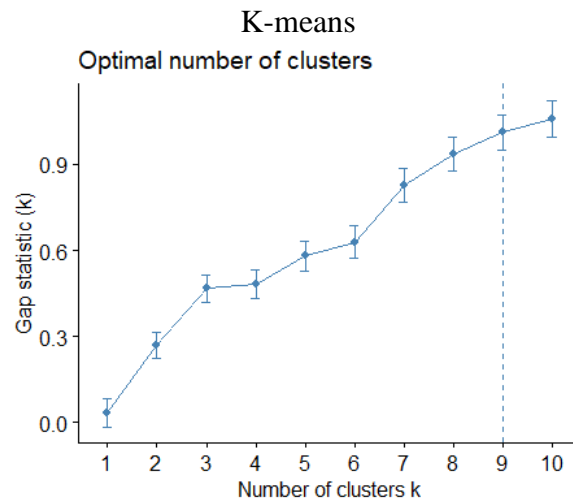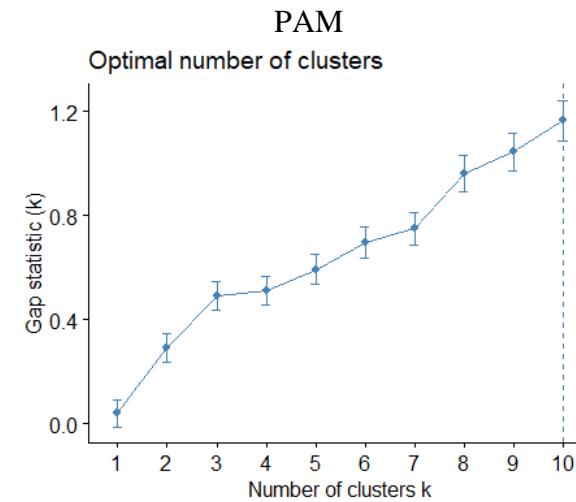

### (H1N1) influenza

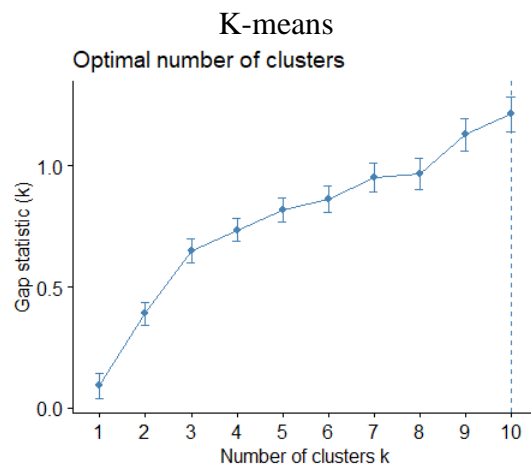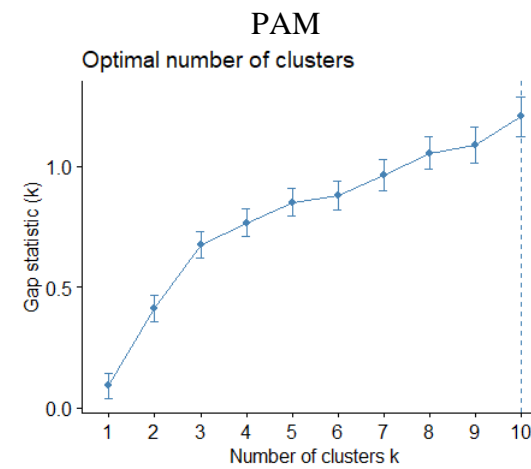

Supplement: Supplementary file 8 — Supplementary Information 8. [file 41598_2022_8574_MOESM8_ESM.pdf]
