# Supplementary material for: Comparing protein–protein interaction networks of SARS-CoV-2 and (H1N1) influenza using topological features
Source: Sci Rep. 2022 Apr 7;12:5867. doi: 10.1038/s41598-022-08574-6 (PMC8988119; doi:10.1038/s41598-022-08574-6)

SARS-CoV-2

Hierarchical

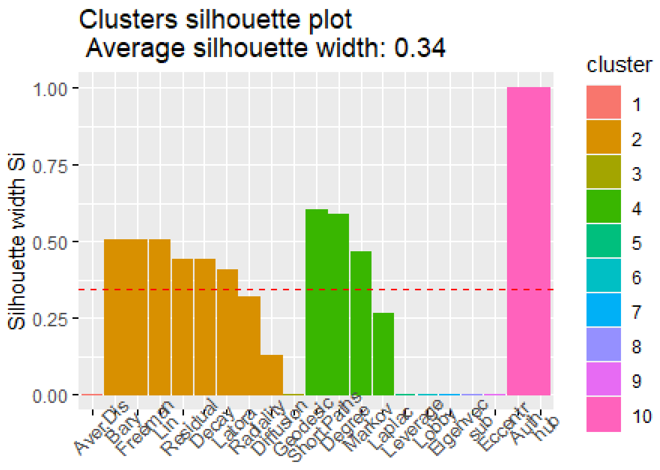

K-means

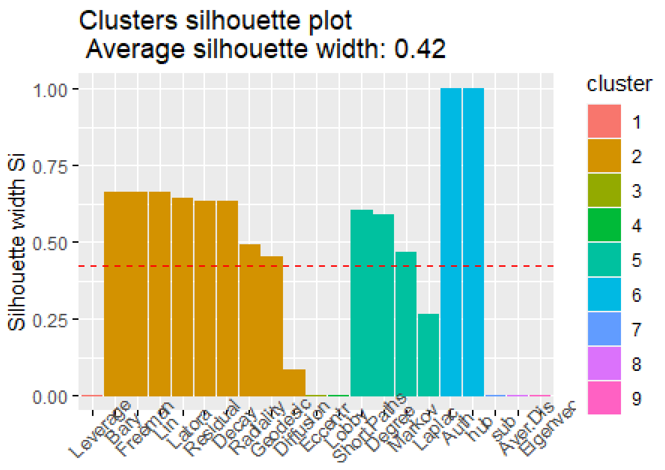

PAM

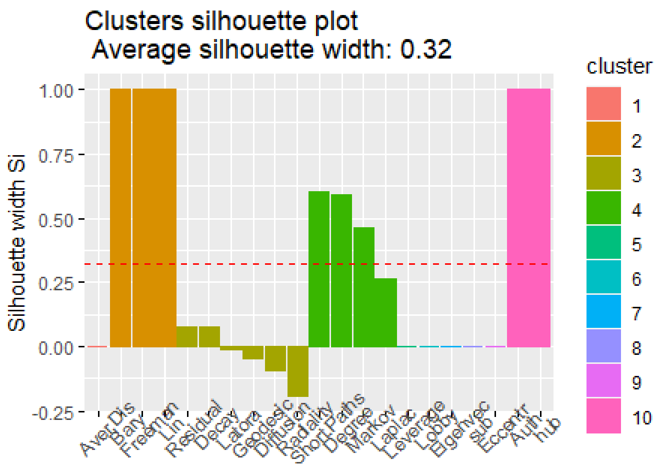

(H1N1) influenza

Hierarchical

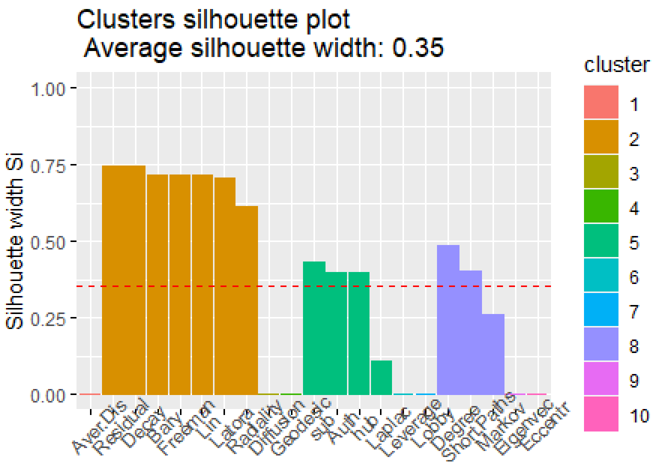

K-means

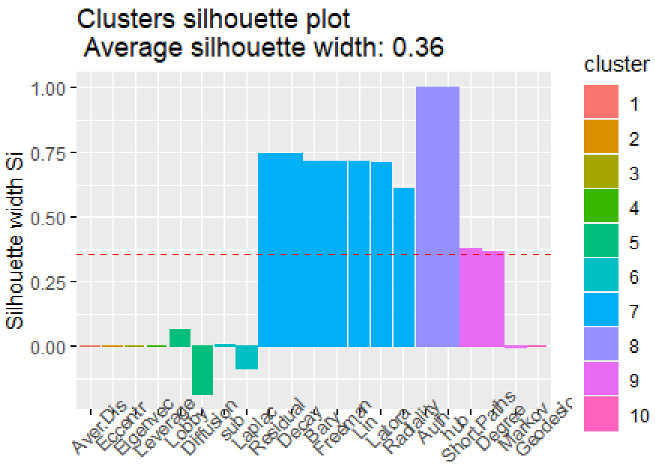

PAM

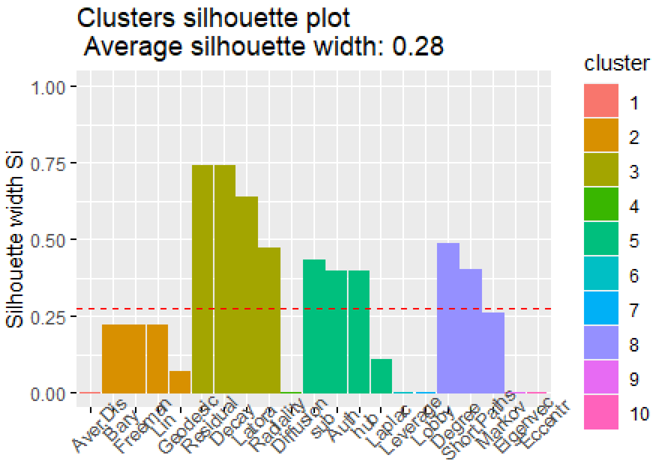

Supplement: Supplementary file 7 — Supplementary Information 7. [file 41598_2022_8574_MOESM7_ESM.pdf]
